# Effect of mangrove leaf extract *(Acanthus ilicifolius)* on non-specific immune status and vibriosis resistance of black tiger shrimps (*Penaeus monodon)* challenged with *Vibrio harveyi*

**DOI:** 10.14202/vetworld.2021.2282-2289

**Published:** 2021-08-31

**Authors:** Gina Saptiani, Slamet Budi Prayitno, Sari Anggarawati

**Affiliations:** 1 Aquatic Microbiology Laboratory, Faculty of Fisheries and Marine Sciences, Mulawarman University, Samarinda 75119, Indonesia; 2 Research Center for Natural Products from Tropical Rainforest (PUI-PT OKTAL), Mulawarman University, Samarinda 75119, Indonesia; 3 Department of Fisheries, Faculty of Fisheries and Marine Science, Diponegoro University, Semarang 50275, Indonesia; 4 Study Program of Agribusiness, Faculty of Agriculture, Nusa Bangsa University, Bogor 16166, Indonesia

**Keywords:** *Acanthus ilicifolius*, herbal plant extract, immunity, *Penaeus monodon*

## Abstract

**Background and Aim:**

There has been continuous effort to search for alternative medicinal plants that are applicable to ameliorate viral disease on shrimp pond. This study aimed to examine the effect of *Acanthus ilicifolius* leaf extract on clinical symptoms and non-specific immune response of black tiger shrimp (*Penaeus monodon*).

**Materials and Methods:**

A total of 330 shrimps were equally assigned into three extract forms (crude extract, ethyl acetate extract, and n-butanol extract, respectively) in which three levels were provided for each extract. Negative control (without leaf extract) and positive control (with oxytetracycline at 0.05 mg/mL) were used, giving a total of 11 experimental treatments.

**Results:**

The results showed that shrimps induced into all form of leaf extracts had significantly higher survival rates, clinical symptoms, and pathological anatomy than those negative control (C−) and positive control (C+). Total hemocyte cells, granulocytes, percentage of phagocytic, and prophenoloxidase activity were similar among leaf extract treatments (p>0.05), but those groups were significantly higher than those of C− and C+ (p<0.05).

**Conclusion:**

n-butanol leaf extract at 300 mg/L is suggested to be the most effective treatment since it showed the highest efficacy on the parameters observed. Thus, it is possible to use the leaf extract of *A. ilicifolius* on-farm as a strategy to enhance bacterial disease resistance and prevent mortality.

## Introduction

In Indonesia, black tiger shrimp (Penaeus monodon) has long been considered as a prized brackish water commodity due to its high economic value and specific market. One province in Indonesia that highly supplies shrimps is East Kalimantan; it is well known to produce organic shrimps [1] and contributes to the largest export value among other commodities [2]. As the demand continues to grow, farmers have steadily increased the density and intensity over the past decade [1]. Unfortunately, shrimps culture intensification is detrimental because it increases the degree of environmental pollution [3] and stressed water [4]. This situation leads to increased mortality in the culture, thereby increasing disease outbreaks that can cause significant economic losses.

However, the intensive use of antibiotics as a therapeutic agent to control the disease adversely impacts the animal, human, and environment with respect to health and sustainability [4-6]. Improvement of shrimp antioxidant and immune capacity is suggested as an effective method to improve surveillance of shrimps in the culture. This is because shrimps rely on the non-specific immune system and possess antioxidant activity to reduce the adverse effect of environmental perturbations [7]. In the past decades, explorations on medicinal plants as a source of growth-promoting agents, antibacterial, and antioxidants have received a growing interest in dealing with the undesirable effect of costly synthetic antibiotics [8,9].

Plants’ bioactive constituents have been acknowledged as effective antibacterial, antiviral, immunomodulator, and other multifunctional purposes [10]. Mangrove plants are abundantly growing in East Kalimantan that have huge potential for the source of novel phytochemical compounds that could be used as a source of pharmaceutical for herbal medicine that is not found in terrestrial plants. Acanthus ilicifolius, one of the mangrove species, grows in the area with low salinity and is being used as traditional medicine.

To the best of our knowledge, phytochemical screening of A. ilicifolius, as well as evaluation of its effect on shrimp immunity, is limited. Therefore, This study aimed to examine the effect of A. ilicifolius leaf extract on clinical symptoms and non-specific immune response of P. monodon.

## Materials and Methods

### Ethical approval

All procedures performed in this study involving shrimps were approved by the Ethical Committee of the Faculty of Fisheries and Marine Sciences, Universitas Mulawarman, Indonesia (Approval certificate number: 078/FKP/2019).

### Study period and location

This experiment was conducted from January to July 2020 in the Laboratory of Aquatic Microbiology, Faculty of Fisheries and Marine Science, Universitas Mulawarman, Samarinda, Indonesia.

### Preparation, extraction, and screening of the phytochemical of *A. ilicifolius* leaf extract

Leaves of *A. ilicifolius* L. var. *xiamenensis* were collected from shrimp culture embankments at Muara Badak District, East Kalimantan, Indonesia. This plant is very well known to the local public. This plant was identified by Iwan Suyatna, a faculty member of the Faculty of Aquaculture and Marine Science, Universitas Mulawarman. Leaves were cleaned and washed with fresh water, drained, and oven-dried. The dried samples were finely sieved to pass a 1 mm screen for phytochemical assays and extraction. The extraction and fraction were concentrated using vacuum distillation [11]. A qualitative test was performed to identify active compounds of *A. ilicifolius* leaf extracts, such as alkaloid, flavonoid, phenol, anthraquinone, anthocyanin, saponin, and tannin, using thin layer chromatography following the methods by Watson [12].

### Bacterial inoculum and pathogenicity test

Bacterial culture was previously isolated from infected *P. monodon* and was prepared according to Xie *et al*. [13]. The pathogens were grown and in a free *Vibrio* culture prepared with seawater at a salinity of 21-23 ppt according to Talpur and Ikhwanuddin [14]. The culture was kept for 6 h after added with 30 mg/L of chlorine and then strongly aerated for 24 h. A 10 mg/L of sodium thiosulfate (Na_2_S_2_O_3_) was added followed by limestone to reach pH 8.00, and then, the supernatant was discharged. Treated seawater was flowed into the filtering tube and added with 5 mg/L ethylenediaminetetraacetic acid (EDTA). Several drops of treated seawater were spread onto TCBSA to test the presence of *V. harveyi*.

### Animals and experimental design

Shrimps were purchased from a commercial hatchery company (CV. Dody Borneo Hatchery) located in Muara Badak District, Kutai Kartanegara Regency, East Kalimantan Province, Indonesia. The broodstocks were healthy and had never been treated by antibiotics, chemicals, nor other medicines. The post-larvae shrimps were cultivated in the grow-out pond. Thereafter, the selected healthy shrimps as indicated from the external appearance and activity were brought to the laboratory, were adapted for 3 days in an acclimated aquarium of 17% salinity with continuous aeration, screened by immersing 100 mg/L formalin for 15 min, and then further adapted for 4 days. The healthy survivors were used for challenge studies.

A total of 330 healthy shrimps were equally distributed into 11 treatments following a completely randomized design with three replicates of each (n=10 per replicate). The treatments consisted of (a) crude leaf extract of *A. ilicifolius* (given at 200, 450, and 700 mg/L concentrations, respectively), (b) ethyl acetate extract of *A. ilicifolius* leaf (at 200, 450, and 700 mg/L concentrations, respectively), and (c) n-butanol extract of *A. ilicifolius* leaf (at 100, 200, and 300 mg/L, respectively). Two other treatments were positive control of antibiotic oxytetracycline at 0.05 mg/mL and negative control of NaCl 0.85%, respectively. Challenge test was conducted according to Prabu et al. [9] on day 7 by injecting 105 CFU/mL of *V. harveyi* using intramuscular injection on the dorsal part because there was evidence that the injection method had a better route to induce infection than immersion. The experiment was performed for 21 days. At the end of the period, survival percentage was calculated by dividing the total number of harvested shrimps by the initial number of shrimps [15].

### Clinical symptoms, anatomical pathology (AP), and hematological analysis

Clinical symptoms were observed by identifying shrimps’ behavior, such as swimming pattern, reflex motion, appetite, and the completeness of shrimps’ bodies. AP was observed on dead shrimps during the period by observing the color changes and organ shape. In addition to the hematological analysis, the sample of hemolymph was collected from the shrimps after 21 days of the experiment using a 1 mL syringe filled with sodium citrate anticoagulation (30 mM trisodium citrate, 338 mM sodium chloride, 115 mM glucose, 10 mM EDTA, and pH 7.0) [9]. The pro-prophenoloxidase (ProPo) assay and total hemocyte cells (THCs) were determined according to Le Moullac *et al*. [16], whereas the nitroblue tetrazolium test (NBT) was performed by the method of Stasiak and Baumann [17] to estimate the respiratory burst activity. In addition, phagocytic cell percentage was calculated by dividing total phagocytes to the phagocytizing, whereas the differential hemocytes (dense and semi-dense granulocytes, hyaline) were determined according to the method of Liu C. and Chen [18].

### Statistical analysis

The data of cellular immunity were subjected to a completely randomized design of analysis of variance and followed by Duncan’s multiple range test when significance was detected at p<0.05 by employing a Statistica 8 software. Residuals were tested for homogeneity of variance and normality. According to the nature of the data, clinical symptom and AP data were analyzed descriptively by comparing means or percentages of the treatments.

## Results

### Phytochemical contents

Screening for phytochemical compounds of crude extract, ethyl acetate extract, and n-butanol leaf extract of *A. ilicifolius* is presented in Table-1. Overall, the leaf of *A. ilicifolius* contains alkaloids, phenol-phenol, flavonoids, and tannins with relatively similar results among the solvent used. The crude extract of the leaf contains a strong level of phenol-phenol and a moderate level of alkaloid and flavonoid. Similarly, n-butanol extract resulted in a strong amount of alkaloid and moderate amount of phenol-phenol, whereas ethyl acetate extract showed a strong effect on flavonoid content. Similar tannins were observed on all extracts.

**Table-1:** Phytochemical content of *Acanthus ilicifolius* leaf extracts.

Parameters	Quantitative analysis of bioactive compounds		

	Crude extract	Ethyl acetate extract	*n-butanol* extract
Alkaloid	+	+	++
Phenol-phenol	++	+	+
Flavonoid	+	++	–
Anthraquinone	–	–	–
Antosian	–	–	–
Saponin	–	–	–
Tannin	+	+	+

+=Mild, ++=Strong

### Survival rates and clinical symptoms

Non-treated shrimps (C−) that were challenged with *V. harveyi* 50% died during the experimental period, whereas the positive control (C+) at treatment of 200 mg/L of crude extract (C1) resulted in 90% survival rates. No mortality was found on the treatment groups of crude extract at 450 and 700 mg/L and on any levels of ethyl acetate as well as n-butanol leaf extracts. In control tanks as a comparison, shrimps showed lethargic, passive, and slow reflex on feed, and red spots were found on the carapace, rostrum, feet, and tail. During the experiment, shrimps on negative and positive controls were unable to molt, indicating that experimental shrimps could not fully recover from bacterial infection until the end of the period (Table-2).

**Table-2:** Survival rate and clinical symptoms of *Penaeus monodon* during the experiment.

Parameters observed, %	Survival rate and clinical symptom (%) of the experimental treatments										

	C1	C2	C3	E1	E2	E3	B1	B2	B3	C–	C+
Survival rate	90	100	100	100	100	100	100	100	100	50	90
Low activity/lethargic	20	-	-	-	-	-	-	-	-	50	20
Low reflex	-	-	-	-	-	-	-	-	-	60	-
Low feeding response	-	-	-	-	-	-	-	-	-	50	20
Red spot at carapace	-	-	-	-	-	-	-	-	-	70	-
Red spot at rostrum	10	-	-	-	-	-	-	-	-	40	20
Red spot at anterior tail	20	-	-	-	-	-	-	-	-	30	20
Red spot at tail	10	20	10	10	-	-	10	-	-	80	10
Red spot at feet	30	10	10	-	-	-	20	-	-	70	40
Tail torn	10	-	-	-	-	-	-	-	-	40	10
Failed molting	20	10	10	10	10	-	10	-	-	60	30

C1=Crude extract at 200 mg/L, C2=Crude extract at 450 mg/L, C3=Crude extract at 700 mg/L, E1=Ethyl acetate fractions at 200 mg/L, E2=Ethyl acetate at fractions 450 mg/L, E3=Ethyl acetate at fractions 700 mg/L, B1=*n-butanol* fraction at 100 mg/L, B2=*n-butanol* fraction at 200 mg/L, B3=*n-butanol* fraction at 300 mg/L, C–=Negative control (PBS), C+=Positive control (with oxytetracycline at 0.05 mg/mL) (antibiotic)

### Pathological anatomy

Most of the pathological anatomies, such as reddish on gills, body, torn tail, rotten tail, abdomen, dorsal cramp, and body deformity, were found to be higher on C− than on other treatments (Table-3). In comparison with C+, shrimps induced with ethyl acetate and n-butanol leaf extracts of *A. ilicifolius* at all levels showed lower pathological damages.

**Table-3:** Average pathological anatomy of *Penaeus monodon* during the experimental period.

Pathological anatomy, %	Pathological anatomy (%) of the experimental treatments										

	C1	C2	C3	E1	E2	E3	B1	B2	B3	C–	C+
Reddish gills	30	20	-	-	-	-	-	-	-	80	30
Damage eye	-	-	-	-	-	-	-	-	-	10	-
Soft flesh	-	-	-	-	-	-	-	-	-	20	20
Reddish on the body	10	-	-	-	-	-	-	-	-	70	20
Dorsal cramp	-	-	-	-	-	-	-	-	-	30	-
Reddish and torn tail	20	20	-	10	10	10	20	-	-	70	-
Reddish rotten tail	30	30	20	10	10	20	20	10	-	90	40
Body deformity	10	-	-	10	-	-	-	-	-	80	10
Red spot at anterior tail	10	10	-	10	-	-	-	-	-	40	20
Reddish at abdomen	10	-	-	-	-	-	-	-	-	60	-
hard abdomen	-	-	-	-	-	-	-	-	-	30	-
Soft brown hepatopancreas	-	-	-	-	-	-	-	-	-	90	-
Shrunken hepatopancreas	-	-	-	-	-	-	-	-	-	40	-

C1=Crude extract at 200 mg/L, C2=Crude extract at 450 mg/L, C3=Crude extract at 700 mg/L, E1=Ethyl acetate fractions at 200 mg/L, E2=Ethyl acetate at fractions 450 mg/L, E3=Ethyl acetate at fractions 700 mg/L, B1=*n-butanol* fraction at 100 mg/L, B2=*n-butanol* fraction at 200 mg/L, B3=*n-butanol* fraction at 300 mg/L, C–=Negative control (PBS); C+=Positive control (with oxytetracycline at 0.05 mg/mL) (antibiotic)

### Hematological parameters

**Figure-1:**
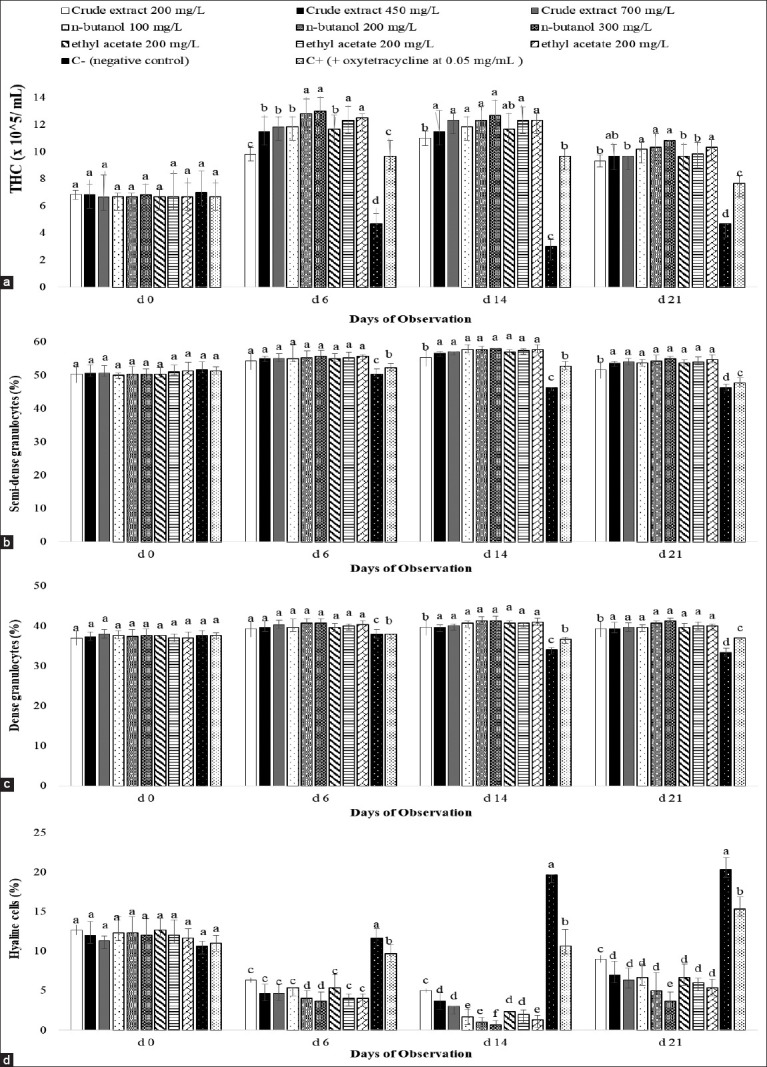
Total hemocytes cells (a), semi-dense granulocyte cells (b), dense granulocyte cells (c), and hyaline cells (d) in plasma of Penaeus monodon-induced different forms of leaf extract of Acanthus ilicifolius. Data are provided as mean±SD (n=3 replicates and 10 shrimp/replicate). Data with different superscripts are significantly different (p<0.05).

**Figure-2:**
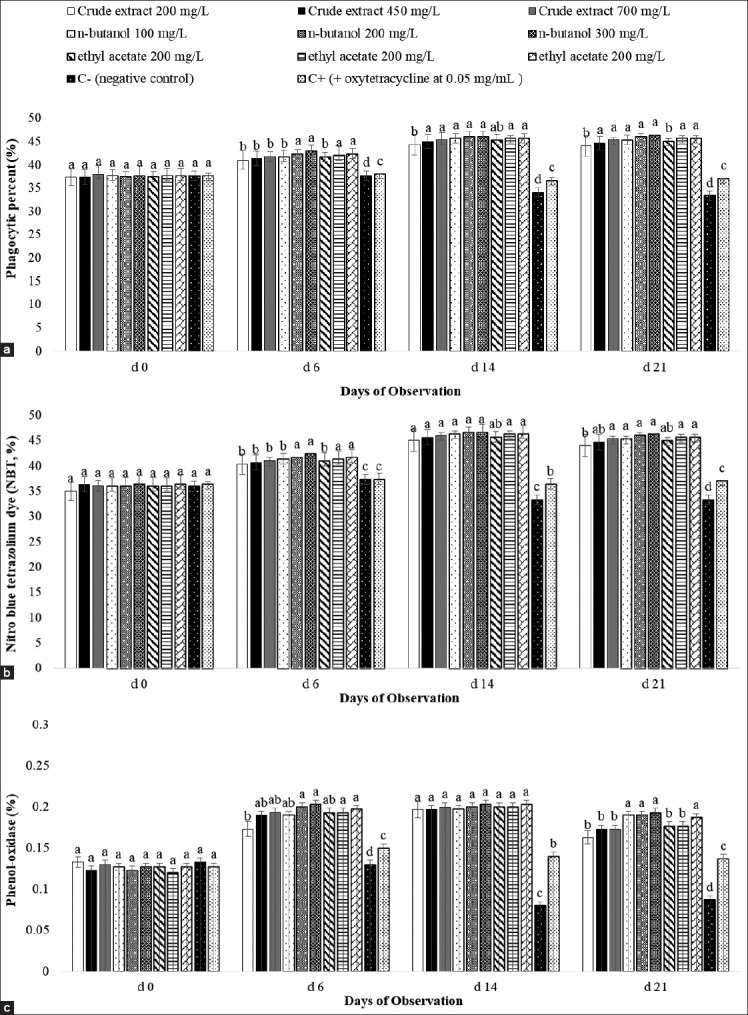
Phagocytic percent (a), nitroblue tetrazolium (b), and phenol-oxidase (c) in plasma of Penaeus monodon-induced different forms of leaf extract of Acanthus ilicifolius. Data are provided as mean±SD (n=3 replicates and 10 shrimp/replicate). Data with different superscripts are significantly different (p<0.05).

THCs in all treatments were similar on day 0 (p>0.05; Figure-1). From day 6 to the end of the experiment, the trends shared a similar pattern for THC, dense granulocytes, semi-dense granulocytes, phagocytosis, nitroblue tetrazolium, and ProPo, whereas the negative control and positive control groups were found to be significantly lower (p<0.05; Figure-2). Shrimps treated with n-butanol given at any levels and ethyl acetate given at the highest level (700 mg/L) were significantly higher on THC and ProPo than those of other treatments (p<0.05). Overall, there were significant effects of extract treatments compared with the control groups (p<0.05), indicating that the extracts of *A. ilicifolius* had an immunomodulatory effect on *P. monodon*.

## Discussion

It is widely accepted that the application of herbal plant extracts provides multiple benefits for aquatic animals, including shrimps, such as improvement on the immune system and antioxidant status [19,20], antibacterial function particularly [10,14], and survival rate and growth [1,20]. In the present study, we provide evidence that the leaf extract of *A. ilicifolius* contains various bioactive constituents, such as a group of flavonoids, phenol, alkaloids, and tannins. This result confirmed previous phytochemical screening on a variety of mangrove species, such as *Avicennia officinalis* [21], *Suaeda maritime* [22], *Avicennia marina*, *Avicennia germinans*, and *Laguncularia racemosa* [23], which primarily contained flavonoids, alkaloids, and phenolic compounds. Among a large number of species, there is great variability in the number of secondary metabolites due to ethnobotanical factors [22,23].

Extracts with three solvents obtained from different parts of the plant have been found to contain different chemical moieties, such as alkaloids, glycosides, lignins, triterpenoids, saponins, sterols, fatty acids, alkaloids, and also flavonoids [22]. These compounds secreted granulocytes, an active form of ProPo granules, which were converted into ProPo enzyme. The polyphenol compounds are easy to be oxidized and converted from oxidase phenol into quinone oxidase that is able to kill pathogens and can produce melanin [24].

In our study, we found different efficacies among extract forms in protecting shrimps from *V. harveyi* infection. In our previous *in vitro* study, secondary metabolites of *A. ilicifolius* leaf extract effectively inhibited the growth of bacteria and protected the shrimps from *V. harveyi* infection and increased survival [11]. An earlier study of ethyl acetate, ethanol, and methanol extracts of the different parts of *A. ilicifolius* exhibited strong to moderate effects against pathogen infections of shrimp and fish [25]. Crude, ethyl acetate at 400-700 mg/L, and n-butanol extracts at 100-300 mg/L of *A. ilicifolius* leaf possessed growth inhibitor of *V. harveyi* *in vivo*, reducing its prevalences and improving the survival of shrimps [11], which was also confirmed in the present study.

Based on clinical signs and pathological anatomy of experimental shrimps, it was demonstrated that dipping of crude extract improved the health and resistance to *V. harveyi* infection compared to both controls. The prevalence of infected shrimps that were previously administrated with leaf extract showed better survivors. The mangrove leaf extracts were effective at improving the shrimp defense against bacterial diseases and a potential source of metabolites against skin infection diseases [26]. Since shrimps do not have a specific immune system, cellular and humoral immunity plays a crucial role as an innate immune mechanism to defend against pathogens [27]. The results of the present study indicated that crude extract and both fractions were able to stimulate the cellular and humoral immune responses of the experimental shrimps. The increase of THC indicates that *A. ilicifolius* leaf extract enhanced hemocyte cells. Hemocyte is a component of immune cells that play an important role in the process of cellular immunity of shrimps [19,28].

This stimulatory effect was confirmed in this experiment, whereas inducing shrimps with most of the leaf extracts significantly increased the phagocytic percentage, nitroblue tetrazolium concentration, and ProPo activity 6 days after challenged with *V. harveyi* to the end of the experiment compared to the C− and C+ groups (day 21) (Figure-2). This result was in line with previous reports that feeding herbs to challenged *P. monodon* significantly increased ProPo activity [9,29]. ProPo is an important innate immune system that plays an important role to protect invertebrates from microbial infection. ProPo is also involved in the acute phase of *Vibrio alginolyticus* infection in *Litopenaeus vannamei* [30].

Differential hemocytes were performed in accordance with Saptiani *et al*. [1]. Semi-dense granulocytes were pink with granulocytes in the center (Figure-1). These cells are involved in the phagocytosis process of shrimp antigen. An increase of hemocyte cells in the present study is a clear indicator that mangrove leaf extract acts as an immunostimulator as previously explained [31]. Granules in the hemocyte cells contained prophenoloxidase precursor [32]. The presence of antigen would activate the protein binding of hemocyte outer layer cells. This process stimulated the release of prophenoloxidase enzyme from the granules in the hemocyte cells.

The increase of phagocytosis was in line with the increase of total hemocyte, semi-dense, and dense granulocyte cells. Phagocytosis is part of hemolymph in the invertebrate defense system, and it acts as an early internal defense mechanism against invaders by circulating hemocytes [20]. The increase of NBT in this experiment suggested that hemocyte cells increased the production of superoxide. The NBT was used to determine superoxide production as a response to stimulate phagocytosis [9].

## Conclusion

The present study concluded that the leaf extract of *A. ilicifolius* is effective in ameliorating the pathogenicity effect of *V. harveyi* as the extracts reduced mortality and clinical symptom and improved the cell immunity of *P. monodon*. In addition, n-butanol leaf extract at 300 mg/L is suggested to be the most effective treatment since it showed higher efficacy than that of antibiotics and other treatments in improving cellular and humoral immune status. No mortality and clinical symptoms were recorded in this treatment. Thus, it is possible to use the leaf extract of *A. ilicifolius* on the farm as a strategy to enhance bacterial disease resistance and prevent mortality.

## Authors’ Contributions

GS and SBP: Designed and conceptualized the experiment. GS and SA: Performed the experiment, laboratory analysis, data analysis, and data curation. GS: Drafted the manuscript. SA and SBP: Reviewed the manuscript. All authors read and approved the final manuscript.
